# Serum Zinc Threshold and the Prognostic Impact of Zinc Supplementation in Liver Cirrhosis

**DOI:** 10.3390/nu18091479

**Published:** 2026-05-06

**Authors:** Yuki Tanaka, Nobuharu Tamaki, Hiroyuki Nakanishi, Takuya Shima, Mina Taguchi, Yudai Yamazaki, Naoki Uchihara, Risa Seike, Shohei Kimura, Junko Yagita, Ryohei Kobayashi, Yuka Kasano, Yasuyuki Komiyama, Kenta Takaura, Hitomi Takada, Shohei Tanaka, Chiaki Maeyashiki, Yutaka Yasui, Kaoru Tsuchiya, Yuka Takahashi, Namiki Izumi, Masayuki Kurosaki

**Affiliations:** Department of Gastroenterology and Hepatology, Musashino Red Cross Hospital, Tokyo 180-8610, Japan; yuki.tanaka.lcpht@gmail.com (Y.T.);

**Keywords:** zinc, cirrhosis, chronic liver disease

## Abstract

**Background and Aim:** Hypozincemia is common in patients with liver cirrhosis; however, its impact on prognosis and the prognostic cutoff remain unclear. This study aimed to identify a prognostic serum zinc threshold using data-driven methods and to evaluate the prognostic association of zinc supplementation with overall survival (OS). **Methods:** Among 721 zinc treatment-naive patients with liver cirrhosis, a prognostic serum zinc threshold associated with OS was determined. OS was subsequently compared between zinc-treated and untreated patients with baseline serum zinc below this threshold. Baseline characteristics were balanced between the zinc-treated and untreated groups (*n* = 119 each) using propensity score matching (PSM). **Results:** Analyses examining the association between serum zinc levels and prognosis demonstrated a dose-dependent relationship with OS. A serum zinc level of 70 µg/dL was identified as a prognostic cutoff strongly associated with OS. Among patients with serum zinc levels <70 µg/dL after PSM, the median OS was longer in the zinc-treated group than in the untreated group (86.4 vs. 47.5 months; *p* = 0.034). The adjusted hazard ratio (95% confidence interval) for OS in the zinc-treated group compared with the untreated group was 0.64 (0.44–0.94). **Conclusions:** A prognostic serum zinc threshold was identified in patients with liver cirrhosis, and zinc supplementation was associated with improved survival. Routine monitoring and zinc supplementation to maintain levels above this reference value may contribute to better prognosis. However, due to the retrospective design and potential residual confounding, future prospective trials are required to validate these findings.

## 1. Introduction

Chronic liver disease progresses from liver cirrhosis to liver failure, representing a major global health burden [1,2]. Liver cirrhosis-related complications, such as hypoalbuminemia, ascites, esophagogastric varices, and hepatic encephalopathy (HE), significantly impair patient survival and quality of life [3,4].

Zinc is an essential trace element for immune function and protein synthesis [5]. Hypozincemia is highly prevalent in patients with liver cirrhosis and is closely associated with the development of HE and hepatocellular carcinoma (HCC) [6,7,8,9,10,11,12]. Recent meta-analyses and reviews have highlighted the prevalence of hypozincemia in advanced chronic liver disease and the potential therapeutic role of zinc supplementation [13,14]. Although zinc supplementation potentially improves liver function and suppresses carcinogenesis [12,15,16], its impact on long-term prognosis remains controversial, and evidence is not yet firmly established [7,17]. Furthermore, there is no consensus on the prognostic zinc threshold for zinc supplementation. To address this knowledge gap, this study aimed to evaluate whether zinc supplementation is associated with survival benefit of patients with liver cirrhosis and to explore a potential reference value for starting therapy.

## 2. Methods

### 2.1. Study Design and Population

**Figure 1:**
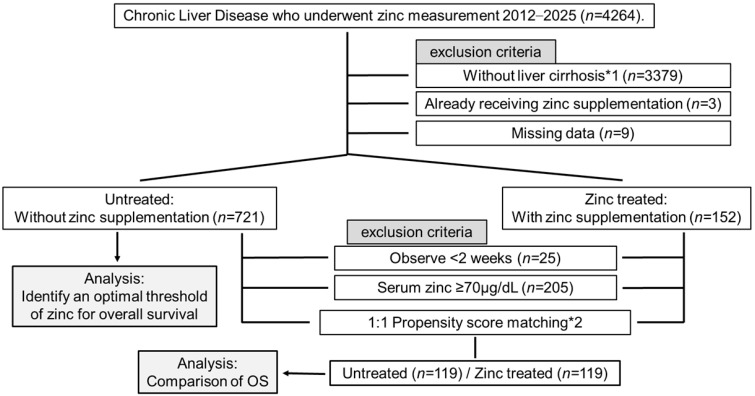
Patients’ flowchart. *1 Patients without liver cirrhosis (e.g., chronic hepatitis, non-cirrhotic portal hypertension, and cryptogenic HCC) were excluded. *2 Propensity score matching was performed adjusting for clinically relevant baseline covariates, including individual liver function parameters (albumin, bilirubin, creatinine, sodium, PT-INR), age, gender, etiology, presence of HCC, ascites, hepatic encephalopathy, and presence of esophagogastric varices. Abbreviations: AIC, Akaike information criterion; HCC, hepatocellular carcinoma; INR, international normalized ratio; OS, overall survival; RCS, restricted cubic spline.

This single-center retrospective observational study was conducted at Musashino Red Cross Hospital. Between 2012 and 2025, 4264 patients with chronic liver disease underwent serum zinc measurement, of whom 885 with compensated or decompensated liver cirrhosis were eligible. We first investigated the association between serum zinc levels and prognosis in untreated patients to identify the potential reference zinc level. Subsequently, we compared outcomes between the zinc-treated group and the untreated group among cases below the identified threshold. Propensity score matching (PSM) was used to balance baseline characteristics between the two groups (Figure 1).

### 2.2. Exclusion Criteria

Patients were excluded based on the following: (1) non-cirrhotic liver disease (*n* = 3379), (2) prior zinc treatment (*n* = 3), and (3) missing data (*n* = 9). The remaining 721 patients were included in the prognostic analysis. For the evaluation of treatment efficacy, we further excluded patients with zinc levels above the identified threshold and those with a follow-up period of less than two weeks (*n* = 25).

### 2.3. Definition of Cirrhosis

Cirrhosis was defined by at least one of the following: (1) histological diagnosis via liver biopsy, (2) endoscopic confirmation of esophagogastric varices, or (3) a clinical history of ascites or HE.

### 2.4. Treatment Protocol

Patients in the zinc-treated group received zinc acetate (98.4%) or zinc histidine (1.6%) at a dosage of 50–100 mg/day as the formulation weight. Following initiation, dosages were adjusted based on periodic serum zinc measurements. Patients with a follow-up period of less than two weeks were excluded from the treatment efficacy analysis. Throughout this manuscript, the term “serum zinc level” refers to the baseline measurement unless otherwise specified.

### 2.5. Primary Outcomes

The primary readout was overall survival (OS). We first established a prognostic zinc threshold in untreated patients and subsequently compared OS between the zinc-treated group and the untreated group among patients falling below this threshold.

### 2.6. Statistical Analysis

To examine the potential non-linear association between baseline serum zinc levels and overall survival, a restricted cubic spline analysis was performed. Three knots were placed at the 10th, 50th, and 90th percentiles of the baseline zinc distribution (40, 58, and 78 µg/dL, respectively). The median zinc level (58 µg/dL) was used as the reference point. The proportional hazards assumption was assessed using Schoenfeld residuals. Regarding the cutoff selection, a detailed AIC-based grid search was conducted at 1 µg/dL intervals. To estimate the uncertainty of the threshold, bootstrap resampling with 1000 iterations was performed to calculate the 95% confidence interval. To compare outcomes between the zinc-treated and untreated groups below this threshold, propensity score matching was performed using nearest-neighbor matching without replacement with a caliper width of 0.2, and exact matching for etiology. The revised PS model directly included independent clinical parameters: age, gender, etiology, baseline serum zinc, albumin, total bilirubin, creatinine, sodium, potassium, C-reactive protein, PT-INR, platelets, Child-Pugh grade, ascites, hepatic encephalopathy, BCAA supplementation, presence of esophagogastric varices, and presence of HCC. In the matched cohort, the impact of zinc supplementation was assessed using the log-rank test and multivariable Cox proportional hazards model. To satisfy the proportional hazards assumption, which was assessed using Schoenfeld residuals, the MELD 3.0 score was categorized by its median (12 points) and used as a stratification factor in the Cox model. Sensitivity analysis was additionally performed using inverse probability weighting (IPW) in the eligible cohort (*n* = 643). The prognostic association of zinc supplementation was estimated using an IPW-adjusted Cox proportional hazards model with robust standard errors. All statistical tests were two-sided, and a *p*-value of <0.05 was considered statistically significant. All statistical analyses were performed using EZR on R commander version 1.68, R commander version 2.9-1, and R version 4.3.1 [18,19,20].

### 2.7. Ethical Considerations

Following approval by the institutional ethics committee, detailed information regarding the study was publicly disclosed on the hospital’s official website and institutional notice boards, ensuring that all patients were guaranteed the opportunity to opt out. In strict accordance with national ethical guidelines for medical research, deceased patients were also included in the opt-out protocol.

### 2.8. Safety Monitoring and Definition of Copper Deficiency

In this retrospective study, safety was assessed through routine clinical visits, physical examinations, and blood tests typically scheduled at least every 3 months. Serum copper levels were monitored at the physician’s discretion. Copper deficiency was clinically defined as a serum copper level <70 µg/dL accompanied by unexplained anemia, leukopenia, or neurological symptoms.

## 3. Results

### 3.1. Patient Characteristics

Table 1 summarizes the baseline characteristics of the 721 zinc treatment-naive patients. The mean age was 69.7 ± 11.2 years, and 449 (62.3%) were male. The primary etiologies were hepatitis C virus (40.9%), followed by alcohol-related liver disease (29.1%) and metabolic dysfunction-associated steatotic liver disease (15.4%). HCC was present in 397 patients (55.1%). Regarding liver function, 252 patients (35.0%) were Child-Pugh class B and 43 (6.0%) were class C. Esophagogastric varices were distributed as follows: F0 in 18.7%, F1 in 25.5%, and F2/F3 (indicated for treatment, including prophylactic cases) in 55.8% (F2: 37.4%, F3: 18.3%) of the patients. The mean baseline serum zinc level was 59.4 ± 16.0 μg/dL.

### 3.2. Target Serum Zinc Levels for Clinical Intervention

**Figure 2:**
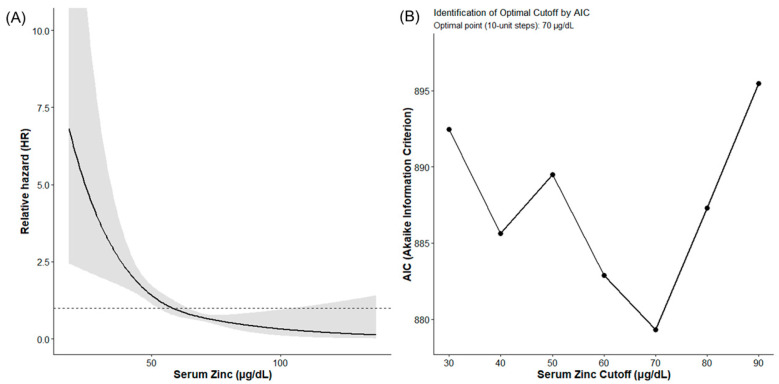
Association between serum zinc levels and prognosis. Association between baseline serum zinc levels and mortality risk. (**A**) Restricted cubic spline (RCS) curve illustrating the relationship between baseline serum zinc levels and the hazard ratio for mortality. (**B**) Akaike Information Criterion (AIC) values derived from a grid search of baseline serum zinc cutoffs in 10-μg/dL increments (ranging from 30 to 90 μg/dL) for predicting overall survival.

**Figure 3:**
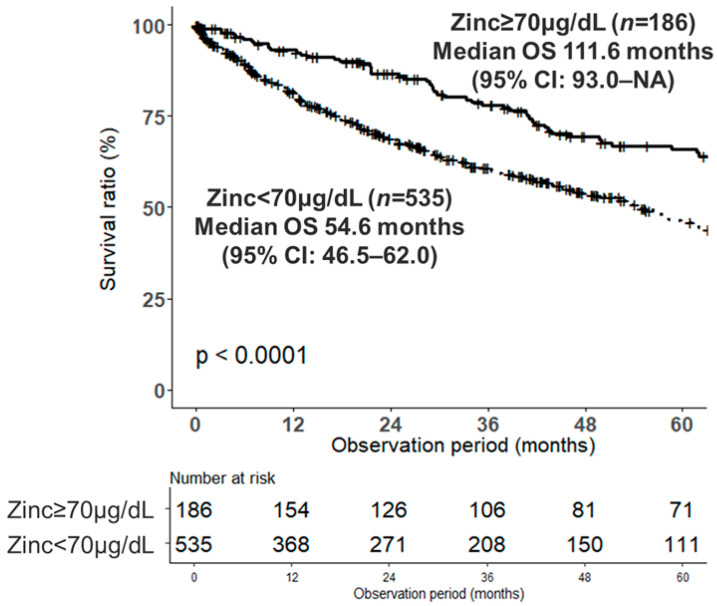
Overall survival by serum zinc levels in the untreated cohort. Kaplan–Meier estimates of overall survival stratified by baseline serum zinc levels (≥70 μg/dL vs. <70 μg/dL) in the untreated cohort. The cumulative OS rates at 1, 3, and 5 years were 93.0%, 78.0%, and 65.9% in the ≥70 μg/dL group (*n* = 186), and 81.7%, 60.8%, and 45.9% in the <70 μg/dL group (*n* = 535), respectively (log-rank test, *p* < 0.0001). Numbers at risk are displayed below the graph.

The RCS model demonstrated a dose-dependent association between serum zinc levels and OS, with mortality risk increasing continuously as zinc levels declined (Figure 2A). The grid search using AIC identified the minimum value at a serum zinc level of 70 μg/dL (Figure 2B). The detailed grid search identified the statistical minimum AIC at 68 µg/dL, with a 95% confidence interval of 54–77 µg/dL determined by bootstrap resampling. Because 70 µg/dL falls within this statistical confidence interval and serves as a practical threshold for daily clinical practice, it was retained as the clinical cutoff for the analysis. To evaluate the dose–response relationship, the cohort was classified by baseline zinc severity (≥70 µg/dL, 60–69 µg/dL, and <60 µg/dL). Kaplan–Meier analysis demonstrated a stepwise worsening of overall survival corresponding to increased severity of zinc deficiency, with median OS of 111.6, 59.8, and 45.2 months, respectively. Pairwise comparisons with Bonferroni correction showed a significant survival difference between the ≥70 µg/dL group and both the 60–69 µg/dL (*p* = 0.015) and < 60 µg/dL (*p* < 0.001) groups. To account for hepatic decompensation status, we performed an additional survival analysis stratified by Child-Pugh class (A vs. B/C). The stratified log-rank test indicated a consistent trend in the prognostic association of the serum zinc threshold across different degrees of liver function (stratified *p* = 0.066). Furthermore, the prognostic association of the 70 µg/dL threshold remained highly significant even after stratifying by presence of HCC (stratified log-rank test, *p* < 0.001); for instance, within the HCC cohort, the median OS was 68.4 months in the ≥70 µg/dL group compared to 37.7 months in the <70 µg/dL group. Based on these findings, we defined 70 μg/dL as the prognostic cutoff in subsequent analyses. The median OS was significantly longer in the ≥70 μg/dL group than in the <70 μg/dL group (111.6 months [95% CI: 93.0–NA] vs. 54.6 months [95% CI: 46.5–62.0]; log-rank *p* < 0.0001; Figure 3).

### 3.3. Propensity Score Matching

To evaluate the prognostic association of zinc supplementation, 1:1 PSM was performed for patients with zinc levels <70 μg/dL. After matching, 119 patients were included in each group. Overlap assessment confirmed an adequate common support region, as visualized in the density plot of propensity scores (Figure S1). Regarding covariate balance, the SMDs for key prognostic variables—including age, etiology, presence of HCC, liver function parameters, and baseline serum zinc levels—were <0.20 (Table 2).

### 3.4. Difference in Overall Survival

**Figure 4:**
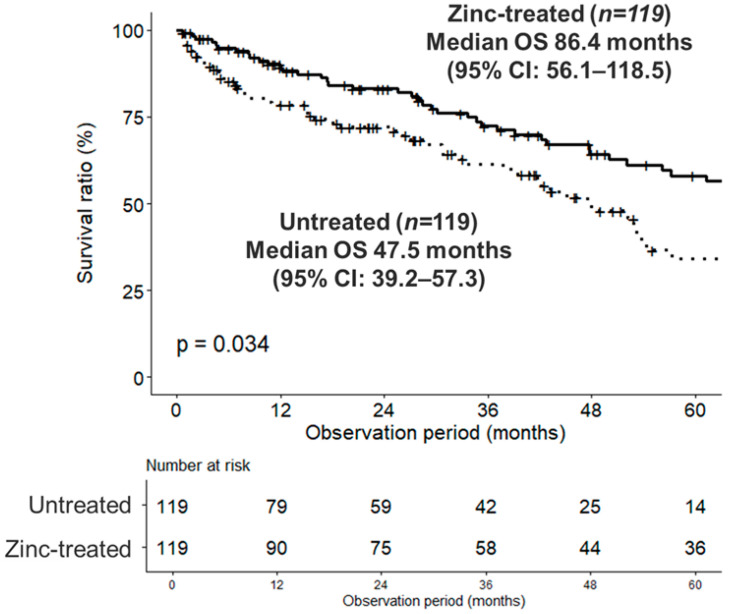
Overall survival in two groups: Zinc-treated and Untreated. Kaplan–Meier estimates of overall survival comparing the zinc-treated and untreated groups. Statistical comparison was performed using the log-rank test. Numbers at risk are displayed below the graph.

**Figure 5:**
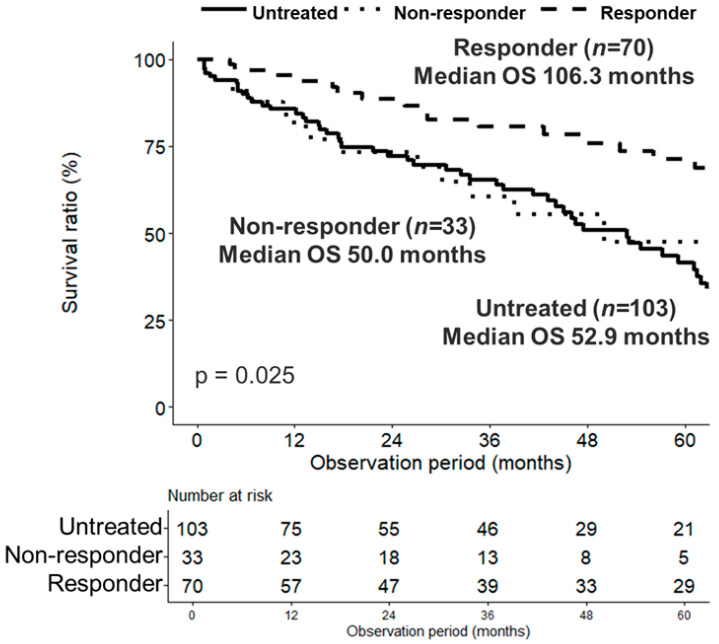
Overall survival based on target zinc level achievement (Responder analysis) in the propensity score-matched cohort. Kaplan–Meier estimates of overall survival comparing Responders (patients who achieved a serum zinc level ≥70 µg/dL during follow-up, *n* = 70), Non-responders (patients who failed to achieve ≥70 µg/dL despite zinc supplementation, *n* = 33), and the untreated group (*n* = 103). Statistical comparison was performed using the log-rank test. Numbers at risk are displayed below the graph.

In the PSM-adjusted cohort, we compared OS between the two groups (Figure 4). The median OS was significantly longer in the zinc-treated group (*n* = 119) than in the untreated group (*n* = 119) (86.4 months [95% CI: 56.1–118.5] vs. 47.5 months [95% CI: 39.2–57.3]; *p* = 0.034). To evaluate whether survival tracks with biochemical correction, an exploratory responder analysis was conducted within the propensity score-matched cohort (*n* = 206). responders (achieved zinc ≥70 µg/dL, *n* = 70) demonstrated a significantly longer overall survival compared to the untreated group (*n* = 103) (Median OS: 106.3 vs. 52.9 months; adjusted *p* = 0.025, Bonferroni correction). Conversely, the non-responder group (failed to achieve ≥70 µg/dL, *n* = 33) showed no survival benefit compared to the untreated group (Median OS: 50.0 vs. 52.9 months; adjusted *p* = 1.000) (Figure 5).

### 3.5. Prognostic Association of Zinc Treatment with Overall Survival in Patients with Liver Cirrhosis

**Figure 6:**
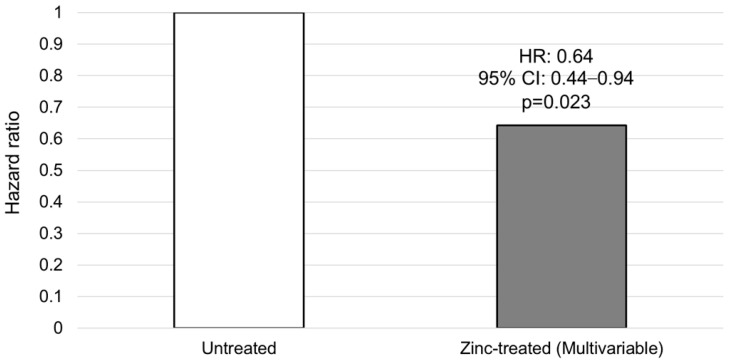
Hazard ratio for mortality associated with zinc supplementation. Multivariable Cox proportional hazards regression analysis comparing the zinc-treated group to the untreated group (reference). The model was adjusted for the following covariates: age, presence of HCC, ascites, hepatic encephalopathy, and presence of esophagogastric varices, and was stratified by the MELD 3.0 score.

In the multivariable stratified Cox proportional hazards analysis, zinc supplementation was significantly associated with a reduced risk of mortality (Hazard Ratio [HR]: 0.64; 95% CI: 0.44–0.94; *p* = 0.023). This model was adjusted for age, presence of HCC, ascites, hepatic encephalopathy, and presence of esophagogastric varices, and was stratified by the median MELD 3.0 score (12 points) to satisfy the proportional hazards assumption (global Schoenfeld test, *p* = 0.395). As a sensitivity analysis, IPW-adjusted Cox regression was performed in the eligible cohort (*n* = 643), which yielded consistent results (Adjusted HR: 0.64; 95% CI: 0.45–0.91; *p* = 0.013) (Figure 6).

### 3.6. Safety and Tolerability

During the follow-up period, no serious adverse events or symptomatic copper deficiencies were observed under this protocol.

## 4. Discussion

### 4.1. Summary of Main Findings

This study demonstrated a clear dose-dependent association between serum zinc levels and mortality risk in patients with liver cirrhosis. We identified 70 μg/dL as a clinically relevant prognostic cutoff; zinc supplementation in patients below this threshold was associated with longer overall survival. Furthermore, survival association were prominently observed in patients who successfully achieved the target zinc level (≥70 µg/dL) during treatment (Figure 6).

### 4.2. Interpretation and Potential Mechanisms

Zinc deficiency is highly prevalent in liver cirrhosis and contributes to disease progression through impaired ammonia metabolism, progression of liver fibrosis, and immune dysfunction [8,9,21,22,23]. While previous studies have shown that zinc supplementation improves HE, reduces ammonia levels, and preserves liver function [7,12,15,24], its impact on long-term survival has remained controversial [17,25]. While current clinical guidelines for liver cirrhosis emphasize nutritional management, they lack specific quantitative targets for zinc intervention [26]. Existing nutritional guidelines, such as those from the Japanese Society for Clinical Nutrition, typically define overt zinc deficiency as <60 µg/dL and marginal deficiency as 60–80 µg/dL based on descriptive biochemical parameters [27]. Meanwhile, the International Zinc Nutrition Consultative Group (IZiNCG) suggests 70 µg/dL as the cutoff for deficiency in the general population [6]. In contrast, this study employed a survival-based predictive approach. Our findings indicate that a prognostic inflection point for mortality risk lies near 70 µg/dL. This suggests that levels traditionally classified as a “marginal deficit” are already associated with decreased overall survival. The observed survival association likely results from a combination of mechanisms, including hepatocyte protection via anti-inflammatory and antioxidant effects [5,28], enhanced immunity through restored T-cell function [29,30], and potential suppression of carcinogenesis [11,15,16].

### 4.3. Clinical Implications

These findings indicate that serum zinc levels are significantly associated with overall survival. Therefore, zinc may serve as a clinically relevant prognostic biomarker for identifying high-risk patients in the setting of liver cirrhosis. Thus, rather than being a purely descriptive nutritional label, 70 µg/dL may serve as a potential reference value in clinical practice. Our results suggest that clinicians might consider proactive intervention at the marginal deficiency stage, as maintaining serum zinc levels above this threshold may be associated with an improved patient prognosis.

### 4.4. Strengths and Limitations

This study has strengths, such as the data-driven identification of the prognostic cutoff using RCS and AIC, alongside rigorous PSM, stratified Cox, and IPW analyses. However, due to the retrospective, single-center nature of this study, several constraints must be acknowledged. First, the influence of unmeasured residual confounding factors, such as detailed nutritional status, the presence of sarcopenia, and patient adherence to therapy, cannot be completely excluded. Second, data on certain clinical parameters—including detailed HCC treatment history, specific cancer stages, cancer-related contribution to mortality, and ongoing alcohol consumption—were not systematically available. Third, precise data on the exact timing of treatment initiation, long-term dose intensity, and specific discontinuation dates could not be fully incorporated. Therefore, the risk of calendar-time bias and immortal time bias cannot be completely eliminated, and assessing the precise timing of biochemical improvement remains a limitation. Furthermore, the exploratory responder analysis is inherently susceptible to immortal time bias, as patients must survive long enough to achieve target zinc levels. Finally, as liver transplantation is rarely performed in Japan, no transplant recipients were included in our cohort.

## 5. Conclusions

In conclusion, we identified a prognostic zinc cutoff for patients with liver cirrhosis. Therefore, monitoring serum zinc levels and considering supplementation may be associated with better clinical outcomes. However, prospective validation is required to confirm these observational findings.

## Figures and Tables

**Table 1:** Baseline characteristics of the study cohort.

Characteristic	Value (Overall *N* = 721)
Age, years	69.69 (11.18)
Male sex, *n* (%)	449 (62.3)
Etiology, *n* (%)	
HBV	56 (7.8)
HCV	295 (40.9)
ALD *1	210 (29.1)
MASLD	111 (15.4)
AIH	12 (1.7)
PBC	24 (3.3)
Others *2	13 (1.8)
Presence of HCC, *n* (%)	397 (55.1)
Presence of esophagogastric varices, *n* (%) *3	
F0	135 (18.7)
F1	184 (25.5)
F2	270 (37.4)
F3	132 (18.3)
Child–Pugh grade, *n* (%)	
A	426 (59.0)
B	252 (35.0)
C	43 (6.0)
Child–Pugh score	6.00 [5.00, 7.00]
Ascites, *n* (%)	120 (16.6)
Hepatic encephalopathy, *n* (%)	149 (20.7)
MELD 3.0 score	9.00 [8.00, 12.00]
FIB-4 index	6.01 [4.01, 8.75]
Albumin, g/dL	3.37 ± 0.65
Total bilirubin, mg/dL	1.10 [0.80, 1.60]
Creatinine, mg/dL	0.96 ± 1.13
Sodium, mEq/L	139.02 ± 3.16
Potassium, mEq/L	4.15 ± 0.50
C-reactive protein, mg/dL	0.14 [0.05, 0.41]
Ammonia, μg/mL	50.50 [35.00, 72.00]
PT-INR	1.11 [1.04, 1.21]
Platelets, 10^9^/L	96.00 [71.00, 128.00]
Serum zinc level, μg/dL	59.40 ± 16.01

Data are expressed as median [interquartile range], mean (standard deviation), or number (percentage). The number of missing data points is as follows: C-reactive protein (*n* = 1), ammonia (*n* = 13), and PT-INR (*n* = 1). All other variables had 100% data completion. HBV, Hepatitis B virus; HCV, Hepatitis C virus; ALD, Alcoholic liver disease, includes one case of metabolic alcoholic liver disease (MetALD) (*1); MASLD, Metabolic dysfunction-associated steatotic liver disease; AIH, Autoimmune hepatitis; PBC, Primary biliary cholangitis; others, includes cases of biliary atresia, hemochromatosis, and cryptogenic (unknown etiology) (*2); F2 and F3 include cases with prior treatment for esophagogastric varices (*3); HCC, Hepatocellular carcinoma; MELD 3.0, Model for End-Stage Liver Disease 3.0; PT-INR, prothrombin time–international normalized ratio.

**Table 2:** Baseline characteristics of the propensity score-matched cohort.

Variable	Before Propensity	Score Matching	SMD	After Propensity	Score Matching	SMD
	Untreated (*n* = 514)	Zinc-Treated (*n* = 129)		Untreated (*n* = 119)	Zinc-Treated (*n* = 119)	
Age (years)	70.05 (11.47)	65.02 (12.36)	0.421	67.11 (11.83)	64.76 (11.96)	0.198
Male sex, *n* (%)	318 (61.9)	78 (60.5)	0.029	78 (65.5)	75 (63.0)	0.053
Etiology, *n* (%)			0.649			<0.001
HBV	27 (5.3)	6 (4.7)		6 (5.0)	6 (5.0)	
HCV	233 (45.3)	26 (20.2)		26 (21.8)	26 (21.8)	
ALD *1	149 (29.0)	66 (51.2)		62 (52.1)	62 (52.1)	
MASLD	72 (14.0)	22 (17.1)		20 (16.8)	20 (16.8)	
PBC	17 (3.3)	8 (6.2)		5 (4.2)	5 (4.2)	
Others *2	10 (1.9)	0 (0.0)		–	–	
Presence of HCC, *n* (%)	300 (58.4)	37 (28.7)	0.627	39 (32.8)	36 (30.3)	0.054
Esophagogastric varices, *n* (%) *3			0.351			0.316
F0	86 (16.7)	33 (25.6)		21 (17.6)	27 (22.7)	
F1	139 (27.0)	24 (18.6)		28 (23.5)	23 (19.3)	
F2	185 (36.0)	56 (43.4)		42 (35.3)	53 (44.5)	
F3	104 (20.2)	16 (12.4)		28 (23.5)	16 (13.4)	
Child–Pugh grade, *n* (%)			0.429			0.091
A	260 (50.6)	41 (31.8)		46 (38.7)	41 (34.5)	
B	217 (42.2)	67 (51.9)		58 (48.7)	61 (51.3)	
C	37 (7.2)	46 (35.7)		15 (12.6)	17 (14.3)	
Child–Pugh score	6.00 [6.00, 8.00]	7.00 [6.00, 9.00]	0.457	7.00 [6.00, 8.50]	7.00 [6.00, 9.00]	0.074
Ascites, *n* (%)	94 (18.3)	42 (32.6)	0.417	24 (20.2)	35 (29.4)	0.285
Hepatic encephalopathy, *n* (%)	125 (24.3)	48 (37.3)	0.282	33 (27.7)	43 (36.1)	0.187
MELD 3.0 score	10.00 [8.00, 13.00]	12.00 [9.00, 15.00]	0.406	11.00 [9.00, 14.00]	11.00 [9.00, 15.00]	0.124
FIB-4 index	6.59 [4.50, 9.59]	6.58 [4.63, 10.07]	0.075	6.28 [4.69, 9.18]	6.58 [4.62, 9.95]	0.033
Albumin (g/dL)	3.21 ± 0.59	2.98 ± 0.68	0.361	3.02 ± 0.61	3.04 ± 0.67	0.033
Total bilirubin (mg/dL)	1.10 [0.80, 1.70]	1.40 [0.90, 2.30]	0.34	1.20 [0.90, 2.05]	1.40 [0.90, 2.15]	0.158
Creatinine (mg/dL)	0.92 ± 0.96	0.87 ± 0.51	0.064	0.82 ± 0.31	0.87 ± 0.52	0.133
Sodium (mEq/L)	138.75 ± 3.19	138.27 ± 3.27	0.149	138.50 ± 3.47	138.28 ± 3.10	0.066
Potassium (mEq/L)	4.11 ± 0.50	3.98 ± 0.50	0.263	4.06 ± 0.47	3.99 ± 0.47	0.157
C-reactive protein (mg/dL)	0.16 [0.05, 0.44]	0.26 [0.11, 0.68]	0.255	0.24 [0.10, 0.78]	0.23 [0.11, 0.60]	0.077
Ammonia (μg/mL)	56.00 [38.00, 80.00]	57.50 [38.25, 96.25]	0.159	57.00 [36.00, 75.00]	57.50 [40.50, 94.75]	0.192
PT-INR	1.13 [1.06, 1.25]	1.22 [1.11, 1.33]	0.428	1.16 [1.07, 1.30]	1.21 [1.11, 1.32]	0.169
Platelets (10^9^/L)	92.00 [67.00, 120.75]	89.00 [62.00, 124.00]	0.02	89.00 [65.50, 119.50]	86.00 [60.00, 123.50]	0.087
Serum zinc level (μg/dL)	52.57 ± 10.88	47.83 ± 12.89	0.398	49.15 ± 11.35	49.21 ± 12.01	0.005
BCAA supplementation, *n* (%)	406 (79.0)	122 (94.6)	0.473	113 (95.0)	112 (94.1)	0.037

Data are expressed as median [interquartile range], mean ± standard deviation, or number (percentage). Propensity score matching was performed using nearest-neighbor matching without replacement with a caliper width of 0.2, and exact matching for etiology. The matching model incorporated independent clinical parameters: age, gender, etiology, baseline serum zinc, albumin, total bilirubin, creatinine, sodium, potassium, C-reactive protein, PT-INR, platelets, Child-Pugh grade, ascites, hepatic encephalopathy, BCAA supplementation, presence of esophagogastric varices, and presence of HCC. HBV, Hepatitis B virus; HCV, Hepatitis C virus; ALD, Alcoholic liver disease, includes one case of metabolic alcoholic liver disease (MetALD) (*1); MASLD, Metabolic dysfunction-associated steatotic liver disease; PBC, Primary biliary cholangitis; others, includes cases of biliary atresia, hemochromatosis, and cryptogenic (unknown etiology) (*2); F2 and F3 include cases with prior treatment for esophagogastric varices (*3); HCC, Hepatocellular carcinoma; MELD 3.0, Model for End-Stage Liver Disease 3.0; FIB-4, Fibrosis-4; PT-INR, prothrombin time–international normalized ratio; BCAA, Branched-chain amino acid; SMD, Standardized mean difference.

## Data Availability

The data that support the findings of this study are available from the corresponding author, upon reasonable request due to privacy and ethical restrictions.

## Supplementary Materials

The following supporting information can be downloaded at: https://www.mdpi.com/article/10.3390/nu18091479/s1, Figure S1: Propensity score overlap assessment.
