# Supplementary material for: Serum Zinc Threshold and the Prognostic Impact of Zinc Supplementation in Liver Cirrhosis
Source: Nutrients. 2026 May 6;18(9):1479. doi: 10.3390/nu18091479 (PMC13164721; doi:10.3390/nu18091479)
Supplement: Supplementary file 1 [file nutrients-18-01479-s001.zip › nutrients-4264347-supplementary.pdf]

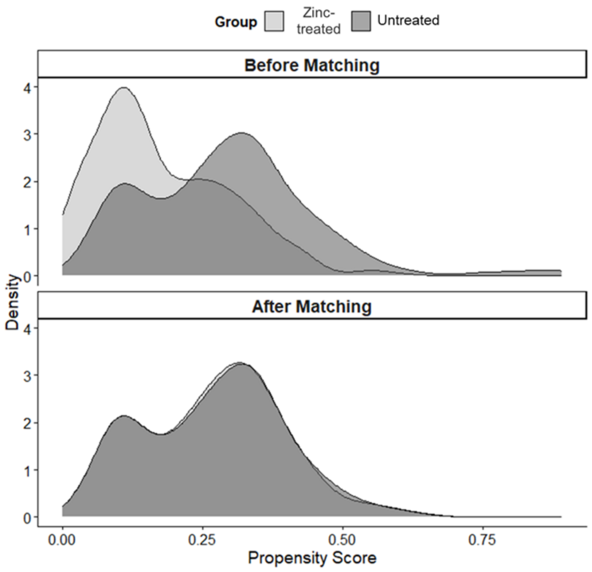

Figure S1. Propensity score overlap assessment. Density plots of propensity scores before matching (upper panel) and after matching (lower panel). The distributions of propensity scores for the zinc-treated group (light gray) and untreated group (dark gray) are shown. The matched cohort was generated using 1:1 nearest-neighbor matching with a caliper width of 0.2 and exact matching for liver disease etiology.
